# The COVID‐19 Pandemic's impact on sustainability and expansion of a Pediatric Early Warning System in resource‐limited hospitals

**DOI:** 10.1002/cam4.5876

**Published:** 2023-04-06

**Authors:** Parima P. Wiphatphumiprates, Dylan E. Graetz, Gia Ferrara, Maria Puerto‐Torres, Srinithya R. Gillipelli, Paul Elish, Hilmarie Muniz‐Talavera, Alejandra Gonzalez‐Ruiz, Miriam Armenta, Camila Barra, Zulma Carpio, Cinthia Hernandez, Susana Juarez, Jose de Jesus Loeza, Alejandra Mendez, Erika Montalvo, Eulalia Penafiel, Estuardo Pineda, Virginia McKay, Asya Agulnik

**Affiliations:** ^1^ Rhodes College Memphis Tennessee USA; ^2^ Global Pediatric Medicine at St. Jude Children's Research Hospital Tennessee Memphis USA; ^3^ Baylor College of Medicine Houston Texas USA; ^4^ Rollins School of Public Health Emory University Atlanta Georgia USA; ^5^ Pediatric Oncology Hospital General de Tijuana Tijuana Mexico; ^6^ Pediatric Oncology Hospital Dr. Luis Calvo Mackenna Santiago Chile; ^7^ Pediatric Oncology Instituto Nacional de Enfermedades Neoplásicas Lima Peru; ^8^ Pediatric Oncology Hospital Infantil Teletón de Oncología Querétaro Mexico; ^9^ Pediatrics Hospital Central Dr. Ignacio Morones Prieto San Luis Potosí Mexico; ^10^ Pediatric Oncology Centro Estatal de Cancerología Xalapa Mexico; ^11^ Pediatric Critical Care Unidad Nacional de Oncología Pediátrica Guatemala City Guatemala; ^12^ Pediatric Critical Care Hospital Oncológico Solca Núcleo de Quito Quito Ecuador; ^13^ Pediatric Oncology Instituto del Cáncer SOLCA Cuenca Cuenca Ecuador; ^14^ Pediatric Oncology Hospital Nacional de Niños Benjamín Bloom San Salvador El Salvador; ^15^ Bloom School of Medicine Washington University St. Louis Missouri USA

**Keywords:** COVID‐19, implementation science, Latin America, pediatric early warning system, pediatric oncology, PEWS, quality improvement collaborative, resource‐limited

## Abstract

**Background:**

The COVID‐19 pandemic impacted healthcare delivery worldwide, including pediatric cancer care, with a disproportionate effect in resource‐limited settings. This study evaluates its impact on existing quality improvement (QI) programs.

**Methods:**

We conducted 71 semi‐structured interviews of key stakeholders at five resource‐limited pediatric oncology centers participating in a collaborative to implement Pediatric Early Warning System (PEWS). Interviews were conducted virtually using a structured interview guide, recorded, transcribed, and translated into English. Two coders developed a codebook of a priori and inductive codes and independently coded all transcripts, achieving a kappa of 0.8–0.9. Thematic analysis explored the impact of the pandemic on PEWS.

**Results:**

All hospitals reported limitations in material resources, reduction in staffing, and impacts on patient care due to the pandemic. However, the impact on PEWS varied across centers. Identified factors that promoted or limited ongoing PEWS use included the availability of material resources needed for PEWS, staff turnover, PEWS training for staff, and the willingness of staff and hospital leaders to prioritize PEWS. Consequently, some hospitals were able to sustain PEWS; others halted or reduced PEWS use to prioritize other work. Similarly, the pandemic delayed plans at all hospitals to expand PEWS to other units. Several participants were hopeful for future expansion of PEWS post‐pandemic.

**Conclusion:**

The COVID‐19 pandemic created challenges for sustainability and scale of PEWS, an ongoing QI program, in these resource‐limited pediatric oncology centers. Several factors mitigated these challenges and promoted ongoing PEWS use. These results can guide strategies to sustain effective QI interventions during future health crises.

## Graphical abstract

The COVID‐19 pandemic created challenges to maintain and scale‐up an ongoing quality improvement initiative, a Pediatric Early Warning System (PEWS), in five pediatric oncology hospitals in Latin America. Identified factors mitigate these challenges and promote ongoing PEWS use can be leveraged to support sustainability of programs to reduce global disparities in childhood cancer during future health crises.

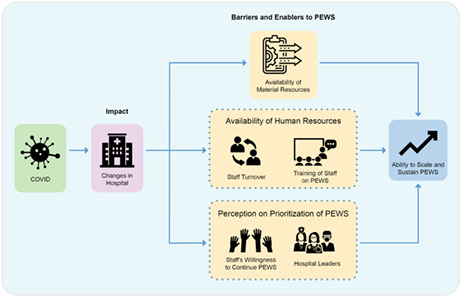

## 1 BACKGROUND

In addition to the direct impact of SARS‐CoV‐2 infection in children with cancer,^1^, ^2^ the COVID‐19 pandemic disrupted healthcare delivery globally,^1^ particularly for pediatric cancer patients in resource‐limited settings such as Latin America.^3^, ^4^, ^5^, ^6^ The pandemic also created challenges to maintain public health programs and ensure access to treatment for all patients.^7^, ^8^ However, there is little research evaluating the pandemic's impact on existing quality improvement (QI) programs in resource‐limited settings.

Proyecto EVAT is an multicenter QI collaborative of pediatric oncology centers in Latin America to implement a Pediatric Early Warning System (PEWS).^9^, ^10^ PEWS are bedside assessment tools composed of a scoring tool and action‐based algorithm for early detection of critical illnesses in hospitalized children.^11^, ^12^, ^13^, ^14^, ^15^ PEWS implementation in pediatric oncology centers has been shown to improve patient outcomes, reduce cost of care, and optimize interdisciplinary communication.^15^, ^16^, ^17^, ^18^, ^19^, ^20^, ^21^ Although sustaining evidence‐based interventions, like PEWS, is important to maintain intervention benefits,^22^ little is known about the impact of healthcare stressors such as the COVID‐19 pandemic on existing QI programs in resource‐limited settings.

Our previous work described barriers to and enablers of PEWS implementation in resource‐limited hospitals prior to the start of the COVID‐19.^23^ Health system disruptions, however, may create additional barriers to continued PEWS use in these settings.^24^ This study evaluates the impact of the pandemic on an ongoing QI program, PEWS, in resource‐limited pediatric oncology hospitals in Latin America and explores factors that impact programmatic sustainability and expansion.

## 2 METHODS

Since all centers completed PEWS implementation prior to March 2020, the start of the COVID‐19 pandemic in Latin America, the primary paper focuses on identifying the barriers and enablers to PEWS implementation pre‐pandemic.^23^ This is a secondary analysis of this data collected to identify challenges toward PEWS sustainability and expansion in resource‐limited hospitals during the pandemic. Details regarding the study methods have been previously reported^23^ and are summarized below. Consolidated Criteria for Reporting Qualitative Research (COREQ) guidelines were followed to ensure rigor of qualitative reporting.^25^

### 2.1 Escala de Valoración de Alerta Temprana (EVAT)

EVAT is a Spanish language PEWS validated in pediatric oncology patients.^26^ Proyecto EVAT is a QI collaborative of resource‐limited pediatric oncology centers in Latin America to implement PEWS.^9^, ^10^ Proyecto EVAT hospitals identify implementation leaders who are trained a unified PEWS implementation strategy and locally administer the program. At the time of this study, all participating hospitals successfully completed PEWS implementation and were focused on sustaining and expanding PEWS to other units (scaling‐up PEWS).

### 2.2 Setting and participants

This study included five Proyecto EVAT hospitals in Mexico, Central, and South America. Participating hospitals were purposefully selected to include centers that implemented PEWS quickly (3–4 months between pilot start and implementation completion) and those that took longer (10–11 months).

Key stakeholder interviews were conducted between June and August 2020. A study lead at each hospital selected approximately 10–15 staff who were PEWS implementation leaders, hospital leaders or directors, or others involved in PEWS implementation to participate in the study.

### 2.3 Human subjects approval

The St. Jude Institutional Review Board approved this study as exempt and minimal risk; therefore, only verbal consent was provided by the participants in the beginning of each interview. Local site leads at each participating center obtained additional approvals as needed.

### 2.4 Interview methods

The study team created a preliminary interview guide, shown in the Figure from the supplemental materials of the primary paper,^23^ to identify barriers and enablers of PEWS implementation based on the Consolidated Framework for Implementation Research (CFIR).^27^, ^28^ As has been done in prior work,^19^, ^29^, ^30^, ^31^ bilingual team members (AG, HT, and MP) translated the interview guide to Spanish and piloted with three individuals not participating in the study but representative of the target population, with revisions made based on feedback. Two bilingual researchers (PE, SG) performed semi‐structured interviews in the participants' native language (Spanish) via Webex. These interviewers were previously unknown to study participants, not employed at participating centers, and not involved in PEWS implementation. Interviews were recorded, transcribed, translated into English, and deidentified prior to analysis.

### 2.5 Analysis

Two authors (AA and GF) developed a codebook based a priori on the CFIR and supplemented by inductive codes identified through iterative review of nine transcripts (Table S1). These researchers independently coded all the transcripts achieving a kappa or intercoder agreement value of 0.8–0.9, meeting with a third researcher (DG) to resolve discrepancies. While there was no interview question specifically asking about COVID‐19, *COVID*, defined as any mention of the pandemic, was identified as an inductive theme from serial review of transcripts. A secondary thematic content analysis focused on identifying challenges hospitals faced in sustaining and scaling PEWS during the ongoing pandemic.

## 3 RESULTS

A total of 71 interviews were conducted in July and August 2020 across five participating hospitals. Participants included those from centers in Lima, Peru (25.4%); San Luis Potosi (SLP), Mexico (15.5%); San Salvador, El Salvador (21.1%); Cuenca Ecuador (21.1%); and Xalapa, Mexico (16.9%). All participants worked in the intensive care unit (ICU) but varied in professions ranging from floor physicians (36.6%), ICU physicians (8.5%), nurses (45.1%), to other hospital‐related professions (9.9%). Participants were majority female (70.4%) with some male (29.6%) and ranged in years working in their center. The categorization of years of experiences participants had in their center are as follows: 0–10 years (38.0%), 11–20 years (35.2%), and 21+ years (26.8%). Specific demographics regarding their roles in PEWS implementation are as follows: PEWS implementation leader (54.9%), PEWS director (29.6%), and other (15.5%).^23^

Participating hospitals were located in San Salvador, El Salvador; Cuenca, Ecuador, Xalapa, Mexico; Lima, Peru; and San Luis Potosi (SLP), Mexico (Table S2). The hospitals specialized in multidisciplinary pediatric care, adult and pediatric oncology, or general adult and pediatric care. All hospitals were public‐funded hospitals, with the exception of the center in Cuenca, Ecuador which was both public and privately funded. Hospitals located in Mexico only contained pediatric ICUs; while the hospitals in Cuenca, Ecuador and Lima, Peru contained an adult ICU.

**FIGURE 1:**
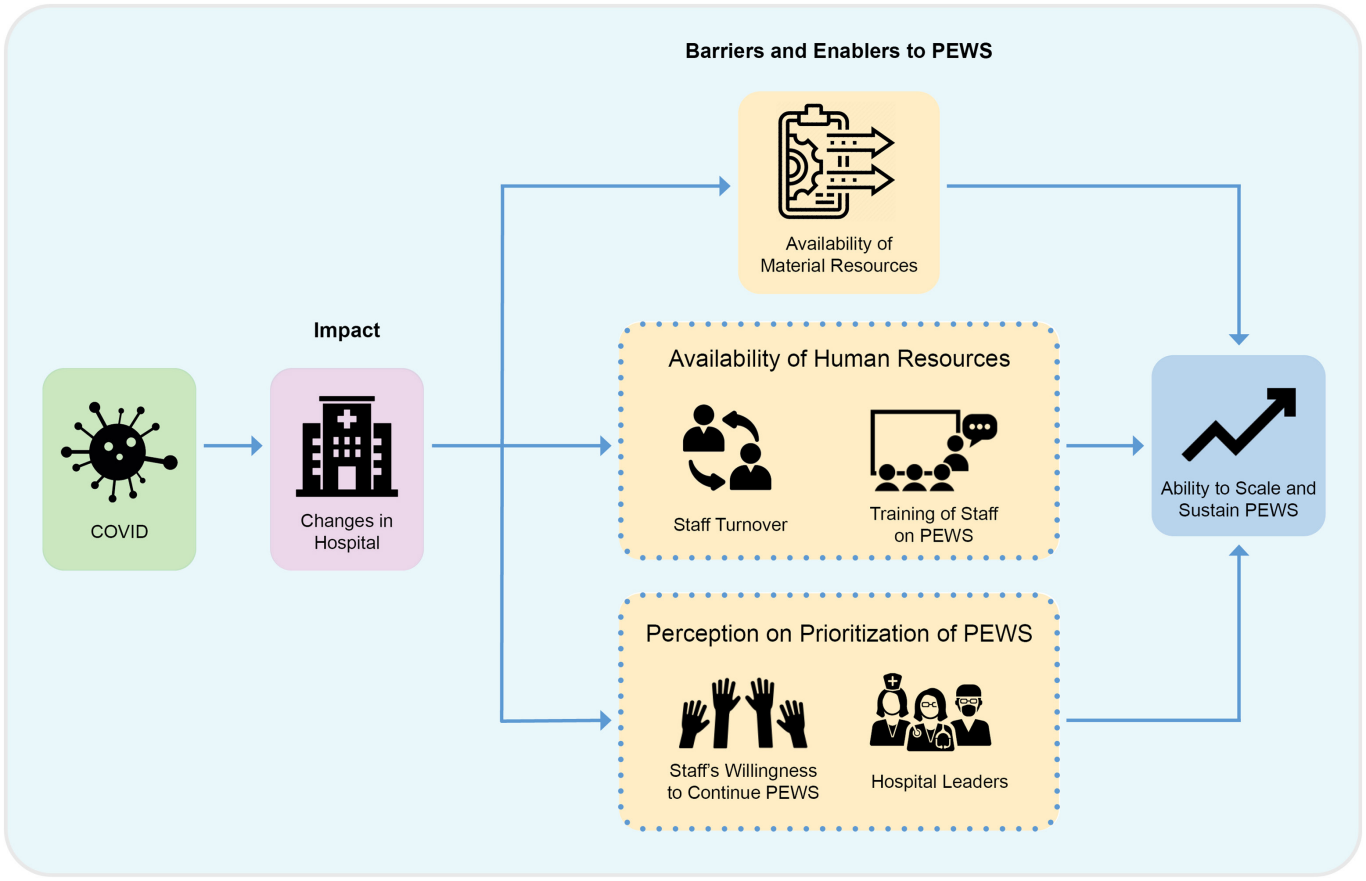
Impact of the COVID‐19 Pandemic on Pediatric Early Warning System (PEWS) Sustainability and Scale. This figure describes the impact of the COVID‐19 pandemic on resource‐limited pediatric oncology hospitals and on the ongoing use and expansion of a PEWS. The negative impact of the COVID‐19 pandemic on hospitals was influenced by several factors, including availability of material resources for PEWS, staff turnover, ability to train staff on PEWS, and the willingness of staff and hospital leaders to continue and promote PEWS. These factors ultimately determined whether hospitals were able to sustain and scale PEWS during the pandemic.

Identified themes from analysis included: the *general impact of COVID‐19 on centers*, *factors that influenced ongoing PEWS use during the pandemic*, and the *ultimate impact of the pandemic on PEWS sustainability and scale* (Figure 1).

### 3.1 General impact of the COVID‐19 pandemic on hospitals

In all centers, the COVID‐19 pandemic impacted the hospitals' *availability of material resources*, *availability of staff*, and *patient care or volume* (Table 1).

**TABLE 1:** Impact of COVID‐19 pandemic on hospital centers.

Hospital center	Bed or material resource availability	Availability of human resources	Impact on patient care and volume
INEN (Peru)	This hospital is the main cancer center; we have too many patients… and now with COVID [the number of available beds] has decreased. (Administrator, Lima)	In the service, we are with the human resource…now with the pandemic…the hospital leaders…have reduced my staff. (Nurse Director, Lima)	I think COVID is changing the rhythm of all of our patients, it's changing the life of everybody; patients are infected, in some of them we had to give chemotherapy in those conditions. (Physician Director, Lima)
Solca Cuenca (Ecuador)	Following the famous pandemic of the Chinese virus…our needs have increased a lot, and we always need material resources, economic resources. (Physician Director, Cuenca)	After the pandemic there are less working hours, we have less medical staff, nursing staff, we even have less staff in the kitchen, cleaning department etc. (Implementation Leader, Cuenca)	Because of the pandemic…patients have decreased in the institution. (Nurse Director, Cuenca)
San Louis Potosi (Mexico)	Here in Mexico we are very limited… in equipment, and right now exactly this year…it's been very complicated due to the contingency in general (Implementation Leader, San Louis Potosi)	In this time of pandemic…some of the staff went home, some with morbidity, so right now they have a reduced number of staff in the hospital (Physician Director, San Louis Potosi)	Our hospital restructured. Now we do not have isolated pediatrics there. (Implementation Leader, San Louis Potosi)
San Salvador (El Salvador)	We're still having admissions. We have not stopped treatments. We have not stopped having patients in the hospitalization area. During all of that… we have had the resources for the [ICU] transfers. (Implementation Leader, San Salvador)	We're still having those barriers, because they are not going to be solved, because we still have people missing in the hospital and it's even worse with the pandemic. (Implementation Leader, San Salvador)	In spite of the pandemic we have not had critical patients resulting in death. (Implementation Leader, San Salvador)
Xalapa (Mexico)	We do not take preventing measures because from the hospital's part we are having a hard time to get money for the project with the authorities. (Implementation Leader, Xalapa)	We have a high level of absenteeism in the hospital, the ones that stayed are ready to a leave, so every time we are less people working. (Implementation Leader, Xalapa)	There were more patients, more work; during COVID, in March, April and May, the number of patients decreased on the floor. (Data Manager, Xalapa)

The *availability of material resources* is defined by how hospital resources (including money, physical space, and materials) were affected during the pandemic. Healthcare staff across all centers expressed limitations in the hospital's *material resources*, including hospital beds and economic resources, during the pandemic. Participants in some hospitals mentioned the overflow of hospitalized patients during the pandemic highlighted pre‐existing problems of limited ICU space: “*Our ICU has bed limitations…I'm talking before the pandemic…it's a multi‐use ICU. Even though it's designed for oncology patients, we had to manage that bed limitation to admit both adult patients and pediatric patients*.” (Physician Director, Lima). Additionally, some staff indicated that the pandemic directly caused their hospital to be in “*serious financial conditions*… *due to external factors and not due to a poor administration in the institution*” (Implementation Leader, Cuenca).

Similarly, healthcare staff at all centers noted a reduction in *availability of staff* during the pandemic. Reasons for this included staff being sent home due to preexisting conditions, shortened work hours for nurses, restrictions in the hospital's finances that limited the number of staff able to work, and the removal of students and interns previously supporting patient care (Table 1). Due to these staff shortages, providers who continued to work faced with a heavier workload. In Xalapa, for example, an implementation leader noted this workload was further increased by the need to closely monitor new staff: “*There were a high number of new people with little experience and little ability*.” The impact of the pandemic on staff extended beyond increased workload. An implementation leader in Lima mentioned that some staff also feared the virus: “*And I think it's around the world, with this coronavirus pandemic, it's the same for the patient…maybe both sides are afraid about infections, patient and nurse*.”

While the material and human resources at the hospitals were frequently reduced, the pandemic had varying impacts on *patient care and volume* (Table 1). During the height of the pandemic, participants in some hospitals described increased numbers of patients admitted to their center. However, others described that their hospital's policy change limited patient admissions or patients' inability to access transportation to the hospital reduced patient volume: “*Transportation to the hospital was not allowed and isolation was mandatory*” (Implementation Leader, Cuenca).

### 3.2 Factors that impact the ongoing PEWS use

Although the impact of the pandemic was similar across centers, how it influenced PEWS use varied. Participants described several factors that changed the impact of COVID‐19 on PEWS use, including the *availability of material resources for PEWS*, *staff turnover*, *training* staff on PEWS, *staff willingness to continue PEWS*, and the *role of hospital leaders*. These factors either promoted or limited ongoing use of PEWS during the pandemic (Table 2).

**TABLE 2:** Factors that impact the use of PEWS.

Theme	Example enabler/ positive modifier	Example barrier/negative modifier
Available material resources	Interviewer: Were there enough resources for the implementation of PEWS? Participant: Yes, because this was before the pandemic, yes there were resources and support. (Implementation Leader, Cuenca)	COVID patients are the ones that are taking most of the hospital's budget and basically. (Implementation Leader, San Louis Potosi)
Reduced human resources and staff turnover	For those of us that come daily, we try…so that this program can continue with the same persistence, even though we have fewer residents, even though we do not have interns… I also think that until now PEWS still works well. (Physician Director, San Louis Potosi)	In this moment most of us have entered in a COVID mode… I know there was a critical moment when the nursing staff was reduced due to the quarantine, due to COVID infection. The replacing staff was new so the PEWS categorization was also reduced. (Implementation Leader, Lima)
Training of staff on PEWS	That is why despite of the circumstances, the medical and nursing staff is able to continue training. We have our resources and our budget so the staff can be trained. (Administrator, Cuenca)	We had to delay some things we had planned like training sessions…We cannot do it now so that is delayed. (Implementation Leader, San Salvador)
Staff's willingness to continue PEWS	But if I could measure a before and after I think we have not lowered the quality. We keep measuring the errors…The project is developing just as before the pandemic. The staff continues to be committed 100% with the project. (Implementation Leader, San Salvador)	Even with a great interest from the chief of nursing, we are trying to expand it to the rest of areas in the hospital. We could not continue with that due to the pandemic…where I must say some doctors there are not fully convinced, even though the authorities are. (Implementation Leader, Xalapa)
Role of hospital leaders	They support us… They have allowed us to install PEWS…It is been working now and we are going to install it for the COVID patients in oncology too…. Because now we are trying to restructure PEWS in a different or COVID area. (Implementation Leader, Lima)	But with the change [in leadership] and now with the pandemic, we have had difficulties because PEWS is not seen as a priority. In this moment the priority is to fight the pandemic. (Implementation Leader, San Salvador)

Some hospitals expressed that they had sufficient resources to continue using PEWS despite the pandemic; however, others noted that maintaining PEWS usage became more difficult when their economic situation deteriorated. For instance, obtaining enough vital sign equipment to properly evaluate patients allowed staff to continue PEWS: “*We solved [the barrier] and…finally overcame that barrier. We got high‐tech monitors to take vital signs*” (Implementation Leader, San Salvador). Contrastingly, the lack of sufficient material resources and financial strain due to allocation of resources for COVID‐19 patients contributed to other hospitals' inability to maintain PEWS use. For example, one hospital used existing space for COVID‐19, rather than pediatric oncology patients, reducing PEWS use: “*Unfortunately, the service where the PEWS pilot started has disappeared, at the moment the area is used to treat COVID‐19 pediatric patients*” (Nurse director, San Louis Potosi).

Similarly, the pandemic's impact on high *staff turnover* (or rotation new staff in the unit) and decreased *availability of staff* dedicated to maintaining PEWS influenced the hospitals' ability to continue using PEWS. Some participants expressed that they were able to adapt to reductions in staffing and redistribute existing workload, limiting impact on continued PEWS use. In other hospitals, however, staff reduction and COVID‐19 restrictions discouraged PEWS usage when introducing staff who were not previously trained in PEWS: “*We tried to make PEWS apply to all children… but then COVID‐19 came and they modified a lot of things…lot of nurses got medical leave and they sent us new staff who were not trained so it turned out very difficult*” (Implementation Leader, San Louis Potosi). Additionally, many participants expressed challenges conducting PEWS *training* during the pandemic: *“PEWS usage is currently active but because of the pandemic maintaining PEWS usage is a little bit more difficult since the number of… trainings have decreased”* (Implementation Leader, Lima).

While the availability of resources influenced ongoing PEWS use, the *staff's willingness* (or lack of willingness) *to continue PEWS* also varied across hospitals and influenced whether staff continued to use PEWS during the pandemic. Despite the pandemic, staff in some hospitals showed enthusiasm to continue PEWS: “*Because the staff hasn't lowered their guard and the staff continues to train themselves*” (Physician Director, Cuenca). Similarly, the *hospital leaders'* opinion about and commitment to the PEWS program at the hospital also varied. Important leaders included the unit director, medical director, nursing chief, and other “key” stakeholders. In centers where hospital leaders recognized the positive impact of PEWS and were supportive of the program, PEWS was maintained or expanded: “*The hospital leaders thought PEWS was novelty and wanted to implement it in the entire hospital…I think…that once the pandemic ends, they can retake that and make it successful*.” (Nurse, San Salvador). In other hospitals, staff expressed difficulty engaging hospital leaders who prioritized other initiatives related to the pandemic over PEWS: “*We haven't been able to meet with the general director, to it's the most important part because they can help us maintain it*.” (Implementation Leader, Xalapa).

### 3.3 The COVID‐19 pandemic's impact on PEWS sustainability and scale

The *sustainability of PEWS* refers to continued PEWS use long‐term, and the *scale of PEWS* refers to the expansion of PEWS to other units/hospitals after initial implementation. All participants expressed a general need for PEWS at their hospitals and a desire to sustain and scale PEWS to other units, different patient populations, or other hospitals: “*We are always looking for projects to improve the quality of care for pediatric patients. PEWS has been a…very useful tool. It makes nurses' concern more objective…helps the communication between providers… and improves the workflow” (*Implementation Leader, Lima). Participants, however, described variability in factors (*material, human resources, staff's prioritization or PEWS*, etc.) that mitigated the pandemic's impact on PEWS resulted in differences in PEWS sustainability and scale across centers during the pandemic (Table 3).

**TABLE 3:** Impact of COVID‐19 pandemic on sustainability and scale of PEWS.

Theme	Ability to sustain or scale PEWS	Inability to sustain or scale PEWS
Sustainability	We have not had changes in our hospital…The PEWS evaluation has been the same. Children who have needed ICU have gone there in an early way. (Physician Director, Cuenca)	Unfortunately, the pandemic has kind of stopped it because everything is focused on dealing the health crisis we are living…So far there have been no meetings for PEWS. (Implementation Leader, Lima)
	With COVID is another thing. It does influence, but it is still working, it is being applied, it has been just an adjustment we had to do against this situation. (Implementation Leader, Xalapa)	Until now, we have been trying to find some strategies to apply PEWS again to all children but we have not…well, the staff changed and COVID happened. We have not been able to apply PEWS” (Implementation Leader, San Louis Potosi)
Scale	Since the pandemic, we have opened other pediatric area…for children with COVID, so we are looking for the possibility to implement PEWS in those areas…The colleagues that work in that area will help them to identify on time everything we know PEWS does. (Implementation Leader, Lima)	That they would have liked to implement it in other units, but they did not have time. And now they cannot offer any kind of support because they are too busy solving the crisis. (Physician Director, San Salvador)
		We're going to open [PEWS into] a new hospital… but right now due to the pandemic we have not moved (Nurse Director, San Louis Potosi)
Hope for the Future	I think once [the pandemic] is over, we can retake the project and see the fruits of that project's implementation. We already planted the seed [of the project] in the perfect spot with the people that we picked. (Nurse Director, San Salvador)	

Participants expressed variable challenged sustaining high‐quality PEWS use during the pandemic. For instance, the hospital in San Salvador was able to sustain PEWS: “*I don't think we've compromised the quality, we've had one or two patients with deterioration, despite the pandemic I'm still defending this project, and would say we're okay*” (Implementation Leader). For other centers, the pandemic created sustainability challenges: “*At this moment, we're just surviving with PEWS, we're not 100% with PEWS, we're trying not to let it fall down, maintaining it*” (Implementation Leader, Xalapa). At some hospitals, the pandemic changed work priorities and significantly impacted PEWS sustainability: “*Currently, it's very difficult because of the economic situation and now with the pandemic, it's not very flexible. We cannot develop other programs that are not a priority…. COVID‐19 patients are the ones that are taking most of the hospital's budget*” (Implementation Leader, San Louis Potosi).

While the pandemic brought changes that influenced sustainability of PEWS, the pandemic more heavily affected the expansion or scale of PEWS to other units, patient populations, or hospital. In all hospitals, staff mentioned the pandemic interfered with existing plans to expand PEWS: “*Right now with the pandemic everything was delayed, because [PEWS] was ready… but who knows now*” (Implementation Leader, Xalapa). Similarly, prioritization of COVID‐19 patients prevented scaling PEWS: *“The hospital leaders would have liked to implement PEWS in other units but they didn't have time and now they can't offer any kind of support because they are too busy solving the crisis”* (Physician Director, San Salvador). The pandemic also prevented planned PEWS expansion to other hospitals: *“We were going to open a new hospital with PEWS expanded into those units, but right now due to the pandemic, we haven't moved”* (Nurse Director, San Louis Potosi).

In some centers, however, the pandemic highlighted an urgent need to improve patient care, which created an opportunity to scale PEWS to COVID‐19 units: “*Since the pandemic, we have opened…an area for children with COVID‐19. We're looking for the possibility to implement PEWS in those areas, because the colleagues that work in that area will help them to identify on time everything, we know PEWS does*” (Implementation Leader, Lima). PEWS implementation in new units allowed staff to better monitor patients: “*After COVID‐19, it even helps us with the symptoms and the vital signs on every patient. It allows us to make proper decisions*” (Implementation Leader, Cuenca). Additionally, PEWS use in COVID‐19 units allowed staff to transfer patients faster: “*PEWS has helped provide continuous alert to do a good evaluation, good treatment of the patient, and provide new findings of illnesses including the coronavirus. PEWS detected initial and more serious symptoms…which has helped us to isolate patients faster and transfer patients to the ICU or the COVID‐19 unit faster*” (Implementation Leader, San Salvador).

While PEWS expansion was commonly halted during the pandemic, several participants reported plans to expand PEWS in the future: “*The idea is to expand the project to all departments of the hospital. We are waiting for the pandemic to be over because right now it's a little bit difficult due to all the adaptation we've done in the hospital for COVID‐19 patients*” (Implementation Leader, San Salvador). Some participants also anticipated PEWS expansion to other hospitals post‐pandemic: *“Another plan…for the future is to promote the implementation of PEWS in regional hospitals of the state of San Luis Potosi, municipality of Valle, Rioverde, Matehuala. That's a plan we have once this pandemic situation improves*” (Physician Director, San Louis Potosi). All participants, however, emphasized a delay in these plans for expansion due to the pandemic and anticipated more success focusing on the current sustainability the PEWS program.

## 4 DISCUSSION

In this study, we evaluated the impact of the COVID‐19 pandemic on an ongoing QI intervention, PEWS, in resource‐limited hospitals. Despite geographic and organizational differences between participating hospitals, all centers reported similar impacts from the COVID‐19 pandemic on hospital material and human resources, and patient care and volume. The subsequent impact of the pandemic on the PEWS initiative, however, varied across centers, based on factors such as availability of materials needed for PEWS, staff turnover and ability to train staff, and the staff and hospital leaders' willingness to continue PEWS, which determined each hospitals' ability to sustain and scale PEWS despite the pandemic.

While there is little research describing the impact of the pandemic on ongoing QI initiatives in LMICs, studies have demonstrated similar findings of negative impacts on existing public health programs such as the disruption of antiretroviral therapy in China.^7^, ^8^ In high‐income countries (HICs), however, there is evidence that ongoing QI initiatives were more easily maintained during the pandemic due to ongoing availability of needed resources and programmatic support.^32^, ^33^ Identified factors that mitigate pandemic challenges and promote sustainability, such as access to resources supporting PEWS and staff's perception regarding the importance of PEWS, are supported by findings from other literature examining sustainability of QI interventions during the pandemic.^8^, ^32^, ^34^, ^35^

The discrepancy between sustainability of QI programs in HICs and LMICs highlights the vulnerability of initiatives in LMICs to sustainability challenges, especially during external healthcare stressors like pandemics. Findings of this study can guide clinicians and researchers looking to maintain effective QI programs during future stressors that limit resources and staff available for programmatic support. Specific recommendations informed by this work include prioritizing assessment of existing material resources and communicating relevant resource needs to leadership, communicating the benefits and reason for maintaining the program to engage staff and hospital leaders, and proactively identifying and addressing sustainability challenges.^24^

Collaborating internally within hospitals and externally through hospital collaboratives (such as in Proyecto EVAT) can facilitate sharing of strategies to promote maintenance of effective interventions during health crises. Beyond sustainability, this work identified the scale, or expansion of PEWS, as a characteristic of this initiative interrupted by the pandemic. In line with recent research highlighting scale as a new implementation outcome, future work should focus how to operationalize this outcome and if the scale of PEWS was achieved following the pandemic.

This study has several limitations. Although inclusion of only five centers could limit the generalizability of our findings, our robust study qualitative design, diverse selection of participating centers, and in‐depth interviews with a variety of key stakeholders strengthens the credibility of our findings. As in all qualitative studies, this work carries an inherent risk of bias. Since COVID‐19 cases and deaths were low among adult and pediatric cancer patients at the time of the study in the participating countries, forecasting bias is a possible risk. However, available literature from the time the study was conducted suggests that interruptions to pediatric cancer care were more influenced by policies on COVID control rather than actual COVID‐19 cases^3^, ^4^, ^5^, ^6^, ^32^, ^33^ and the impacts of COVID on the sustainability of PEWS was more reflective of this affect. Rigorous qualitative methods were also used to mitigate other potential bias including theory‐driven development of the interview guide with open‐ended interview questions, interview procedures intended to minimize social desirability bias, and use of multiple coders during analysis. Our study similarly only evaluated the pandemic's impact on the sustainability and scale of one intervention at pediatric oncology hospitals, potentially limiting generalizability to other interventions and settings. However, our findings are supported by literature from other settings and can inform global strategies to promote sustainability of other QI programs.^24^, ^34^, ^35^ While this study lays the foundation for future work regarding PEWS, factors influencing long‐term sustainability of interventions like PEWS across large cohort of resource‐limited centers post‐pandemic and its subsequent impact on patient outcomes should be explored in future research.^9^

## 5 CONCLUSION

We present a multicenter, multinational study describing the challenges to sustainability and scale of a QI program, PEWS, during the COVID‐19 pandemic. These findings identify specific factors that can be leveraged to add resilience to existing QI programs and guide strategies to sustain interventions that improve outcomes for pediatric cancer patients during future crises.

## AUTHOR CONTRIBUTIONS

**Parima Prim Wiphatphumiprates:** Formal analysis (lead); visualization (lead); writing – original draft (lead); writing – review and editing (equal). **Dylan E. Graetz:** Conceptualization (equal); formal analysis (equal); visualization (equal); writing – original draft (equal); writing – review and editing (equal). **Gia Ferrara:** Formal analysis (equal); writing – review and editing (equal). **Maria F Puerto‐Torres:** Data curation (equal); formal analysis (supporting); writing – review and editing (supporting). **Srinithya Reddy Gillipelli:** Data curation (equal); formal analysis (supporting); writing – review and editing (supporting). **Paul Elish:** Data curation (equal); formal analysis (supporting); writing – review and editing (supporting). **Hilmarie Muniz‐Talavera:** Data curation (equal); formal analysis (supporting); writing – review and editing (supporting). **Alejandra Gonzalez‐Ruiz:** Data curation (equal); formal analysis (supporting); writing – review and editing (supporting). **Miriam Armenta:** Data curation (equal); formal analysis (supporting); writing – review and editing (supporting). **Camila Barra:** Data curation (equal); formal analysis (supporting); writing – review and editing (supporting). **Zulma Carpio:** Data curation (equal); formal analysis (supporting); writing – review and editing (supporting). **Cinthia Hernandez:** Data curation (equal); formal analysis (supporting); writing – review and editing (supporting). **Maria Susana Juárez Tobias:** Data curation (equal); formal analysis (supporting); writing – review and editing (supporting). **Jose de Jesus Loeza:** Data curation (equal); formal analysis (supporting); writing – review and editing (supporting). **Alejandra Mendez Aceituno:** Data curation (equal); formal analysis (supporting); writing – review and editing (supporting). **Erika Montalvo:** Data curation (equal); formal analysis (supporting); writing – review and editing (supporting). **Eulalia Penafiel:** Data curation (equal); formal analysis (supporting); writing – review and editing (supporting). **Estuardo Pineda:** Data curation (equal); formal analysis (supporting); writing – review and editing (supporting). **Virginia R McKay:** Formal analysis (supporting); supervision (lead); visualization (supporting); writing – review and editing (equal). **Asya Agulnik:** Conceptualization (equal); formal analysis (equal); funding acquisition (lead); supervision (lead); visualization (equal); writing – original draft (equal); writing – review and editing (lead).

## FUNDING INFORMATION

This study was funded by the American Lebanese‐Syrian Associated with Charities (ALSAC). Dr. Agulnik was funded by the Conquer Cancer Foundation Global Oncology Young Investigator Award for this work. These funders were not involved in the design or conduct of the study; collection, management, analysis, or interpretation of the data; preparation, review, or approval of the manuscript; or decision to submit the manuscript for publication.

## CONFLICT OF INTEREST STATEMENT

The authors declare not competing interests.

## TWEET

How did the COVID‐19 pandemic affect sustainability of ongoing quality improvement initiatives in resource limited hospitals? This study identified factors that mitigate pandemic challenges and promote ongoing use of Pediatric Early Warning Systems to improve childhood cancer outcomes.

## Supporting information


**Appendix S1:** Supporting Information

## Data Availability

Parima Wiphatphumiprates, Gia Ferrara, and Asya Agulnik had full access to all the data in the study and takes responsibility for the integrity of the data and the accuracy of the data analysis.
