# Supplementary material for: The COVID‐19 Pandemic's impact on sustainability and expansion of a Pediatric Early Warning System in resource‐limited hospitals
Source: Cancer Med. 2023 Apr 6;12(10):11878–88. doi: 10.1002/cam4.5876 (PMC10242859; doi:10.1002/cam4.5876)
Supplement: Supplementary file 1 — Appendix S1: Supporting Information [file CAM4-12-11878-s001.pdf]

Supplemental Materials

THE COVID-19 PANDEMIC'S IMPACT ON SUSTAINABILITY AND EXPANSION OF THE PEDIATRIC EARLY WARNING SYSTEM (PEWS) IN RESOURCE-LIMITED HOSPITALS

Parima Wiphatphumiprates[1]; Dylan E. Graetz, MD, MPH[2], Gia Ferrara, MSGH[2], Maria Puerto-Torres, BA[2], Srinithya R Gillipelli, BA[3], Paul Elish, MPH[4], Hilmarie Muniz-Talavera, PhD[2], Alejandra Gonzalez-Ruiz, MD, MIH[2], Miriam Armenta, RN[5], Camila Barra, RN[6], Zulma Carpio, RN[7], Cinthia Hernandez, RN[8], Susana Juarez, MD[9], Jose de Jesus Loeza, MD[10], Alejandra Mendez, MD[11], Erika Montalvo, MD[12], Eulalia Penafiel, MD[13], Estuardo Pineda, MD[14], Virginia McKay, PhD[15], Asya Agulnik, MD, MPH[2]

[1]Rhodes College, Memphis, TN
[2]St. Jude Children's Research Hospital, Memphis, TN
[3]Baylor College of Medicine, Houston TX
[4]Rollins School of Public Health, Emory University, Atlanta, GA
[5]Hospital General de Tijuana, Tijuana, México
[6]Hospital Dr. Luis Calvo Mackenna, Santiago, Chile
[7]Instituto Nacional de Enfermedades Neoplásicas, Lima, Perú
[8] Hospital Infantil Teletón de Oncología, Querétaro, México
[9] Hospital Central Dr. Ignacio Morones Prieto, San Luis Potosí, México
[10] Centro Estatal de Cancerología, Xalapa, México
[11]Unidad Nacional de Oncología Pediátrica, Guatemala City, Guatemala
[12] Hospital Oncológico Solca Núcleo de Quito, Quito, Ecuador
[13] Instituto del Cáncer SOLCA Cuenca, Cuenca, Ecuador
[14] Hospital Nacional de Niños Benjamín Bloom, San Salvador, El Salvador
[15] Bloom School of Medicine, Washington University, St. Louis, MO

Corresponding Author:
Asya Agulnik
asya.agulnik@Stjude.org
St. Jude Children's Research Hospital
262 Danny Thomas Place
Mail Stop 721
Memphis, TN 38105

Table of Contents

Supplemental Material	Page
Supplemental Table 1: Code Book	2
Supplemental Table 2: Characteristics of Participating Centers	4
COREQ (Consolidated Criteria for Reporting Qualitative Research) Checklist	5

Supplemental Table 1: Codebook

Code	Definition
Stage of Change	Willingness, or lack of willingness of individuals or authorities **in the hospital** to gain new skills, accept change, or show interest/enthusiasm for EVAT. Includes mentions of resistance to or embracing using EVAT as part of routine patient care, comments about how useful EVAT is/isn't to patient care and other beliefs about the value of EVAT, perceptions of whether EVAT does/does not increase workload, and recommendations to implement EVAT in other centers. Should only be coded when referring to perceptions or actions of individuals in the organization. May be double coded with 'Sustainability' if mentions both individuals and institutional acceptance of EVAT. Does NOT include knowledge or skill on HOW to use EVAT correctly ("skill with EVAT").
Staff Turnover	Any mention of staff turnover, including ration nurses, residents, fellows, physicians, or entry of new staff to the unit. Also includes turn-over of leadership in the hospital. Includes mentions of lack of turnover (staff permanence). If mention of training new staff, double code with "EVAT Training".
Role of Leadership/ Authorities	Commitment, involvement, and accountability of leaders and managers with implementation and use of EVAT, including their role in EVAT. Includes statements like "the leadership was supportive" and giving mandates to do XYZ with EVAT and general awareness about EVAT.
Available Material Resources	The level of resources dedicated to implementation and ongoing use of EVAT, including money, physical space, and materials. This includes monitors and other vital sign equipment as well as EVAT guides or tools, patient boards. Includes mention of how resources were obtained, if mentioning specific resources delegated for this. If the reference is unclear for material vs human resources, code both.
Available Human Resources	The level of human resources dedicated to implementation and ongoing use of EVAT, including personnel and time, or having enough time dedicated for EVAT training (including providing paid time to attend trainings). If the reference is unclear for material vs human resources, code both.
Engaging Staff	Mention of how to attract individuals (nurses, physicians) to implementation and use of EVAT through social marketing, role modeling, and other activities. Any mention of communication about EVAT. Exclude statements related to engaging hospital Leadership or Champions (code separately), mention of training (code "Training") or staff participation in EVAT (code "stage of change" or outcomes" depending on context). If trying to decide between engaging staff and leadership (its unclear) choose this code.
Training	Any mention of training individuals in the hospital on how to use or implement EVAT. Includes mentions of training 1 and 2 and teaching staff how to perform/use EVAT, or re-teaching staff who make mistakes using EVAT.
Engaging Hospital Leaders	Mention of engaging, communicating, or training (or lack of communication with) chiefs and directors in the hospital with respect to implementing and using EVAT. Includes unit director, medical director, authorities, nursing chief, and other hospital leadership, "key stakeholders" and how these individuals were engaged or communicated with to

	participate/support EVAT. Their role or final support (or lack thereof) for implementing/using EVAT should be coded as "Role of Leadership and Authorities." Does not include general statements of hospital leaders being aware.
Scale	Any mention of the expansion of EVAT to other units, hospitals, etc. Any mention of implementing somewhere else after initial implementation. Includes mention of why EVAT can or can't scale up, and how it must be modified to enhance replicability or scale.
Sustainability	The perceived likelihood of continued use of EVAT and activities for the continued achievement of the desired outcomes on patient care, any mention of sustainability or sustainment of use in the long-term, including it becoming part of 'routine' or 'practice' at the hospital.
COVID	Any mention of COVID or the pandemic.
Patient	Any mention of the impact of implementing EVAT in the hospital on patient care, including patient safety, teamwork, communication, and impact on families/parents. Includes negative and positive impacts.

This codebook was adapted from a prior study (*Agulnik et al. The Assessment of Barriers and Enablers to Implementation of Pediatric Early Warning Systems (PEWS) In Resource-Limited Settings: A Qualitative Study and modified to include relevant codes to this study.*

Supplemental Table 2: Characteristics of Participating Centers

Center	Annual New Pediatric Cancer Diagnoses	PHO beds	Average floor nurse: patient ratio (1 nurse for x patients)	Time Required for PEWS Implementation (months)
San Salvador, El Salvador	185	24	6	3
Cuenca, Ecuador	75	22	10	4
Xalapa, Mexico	110	27	4	4
Lima, Peru	800	65	6 to 7	9.9
San Luis Potosi (SLP), Mexico	42	0 (integrated with pediatrics)	6	11.2

Abbreviations: PEWS-Pediatric Early Warning System; PHO-Pediatric Hematology Oncology; SLP-San Luis Potosi

COREQ (Consolidated Criteria for Reporting Qualitative research) Checklist

A checklist of items that should be included in reports of qualitative research. You must report the page number in your manuscript where you consider each of the items listed in this checklist. If you have not included this information, either revise your manuscript accordingly before submitting or note N/A.

Topic	Item No.	Guide Questions/Description	Reported on Page No.
**Domain 1: Research Team and reflexibility**			
Personal Characteristics			
Interviewer/facilitator	1	Which author/s conducted the interview or focus group?	5
Credentials	2	What were the researcher's credentials? E.g. PhD, MD	1
Occupation	3	What was their occupation at the time of the study?	N/A
Gender	4	Was the researcher male of female?	N/A
Experience and Training	5	What experience or training did the researcher have?	5
Relationship with participants			
Relationship established	6	Was a relationship established prior to study commencement?	4-5
Participant knowledge of interviewer	7	What did the participants know about the researcher? E.g. personal goals, reasons for doing the research	4
Interviewer characteristics	8	What characteristics were reported about the interviewer/facilitator? E.g. bias, assumptions, reasons and interests in the research topic	5
**Domain 2: Study Design**			
Theoretical framework			
Methodological orientation and Theory	9	What methodological orientation was stated to underpin the study? E.g. grounded theory, discourse analysis, ethnography, phenomenology, content analysis	5
Participant Selection			
Sampling	10	How were participants selected? E.g. purposive, convenience, consecutive, snowball	4
Method of approach	11	How were participants approached? E.g. face-to-face, telephone, mail, email	5
Sample size	12	How many participants were in the study?	4
Non-participation	13	How many people refused to participate or dropped out? Reasons?	N/A
Setting			
Setting of data collection	14	Where was the data collected? E.g. home, clinic, workplace	5
Presence of non-participants	15	Was anyone else present besides the participants and researchers?	5
Description of sample	16	What are the important characteristics of the sample? E.g. demographic, data, date	5-6
Data Collection			

Interview Guide	17	Were questions, prompts, guides provided by the authors? Was it pilot tested?	5 and Supplement
Repeat Interviews	18	Were repeat interviews carried out? If yes, how many?	N/A
Audio/ visual recording	19	Did the research use audio or visual recording to collect the data?	5
Field Notes	20	Were field notes made during and/or after the interview or focus group?	5
Duration	21	What was the duration of the interviews or focus group?	5
Data Saturation	22	Was data saturation discussed?	4-5
Transcripts Returned	23	Were transcripts returned to participants for comment and/or correction?	N/A
**Domain 3: analysis and findings**			
Data analysis			
Number of data coders	24	How many data coders coded the data?	5
Description of the coding tree	25	Did authors provide a description of the coding tree?	Supplements
Derivation of themes	26	Were themes identified in advance or derived from the data?	5
Software	27	What software, if applicable, was used to manage the data?	2
Participant checking	28	Did participants provide feedback on findings?	N/A
Reporting			
Quotations presented	29	Were participant quotations presented to illustrate the themes/ findings? Was each quotation identified? E.g. participant number	5-9 + Tables
Data and findings consistent	30	Was there consistency between the data presented and the findings?	5-9 + Tables
Clarity of major themes	31	Were major themes clearly presented in the findings?	5, 9-10, Figure 1
Clarity of minor themes	32	Is there a description of diverse cases or discussion of minor themes?	5-9+ Figure 1

Developed from: Tong A, Sainsbury P, Craig J. Consolidated criteria for reporting qualitative work (COREQ): a 32 item checklist for interviews and focus groups. *International Journal for Quality in Health Care.* 2007. Volume 19, Number 6: pp. 349-357
